# Gut health management in livestock: roles of probiotics, prebiotics, and synbiotics in growth, immunity, and microbiota modulation

**DOI:** 10.1007/s11259-025-10927-1

**Published:** 2025-10-20

**Authors:** Md. Rayhan Chowdhury, Mahmudul Hassan, Takeshi Shimosato

**Affiliations:** 1 Graduate School of Medicine, Science and Technology, Shinshu University, Minamiminowa, Nagano, 399-4598 Japan; 2 Department of Genetic Engineering and Biotechnology, University of Rajshahi, Rajshahi, 6205 Bangladesh; 3 Institute for Aqua Regeneration, Shinshu University, Minamiminowa, Nagano, 399-4598 Japan

**Keywords:** Probiotics, Prebiotics, Synbiotics, Domestic animals, Gut health, Productivity

## Abstract

Gut health is increasingly recognized as vital in both human and veterinary medicine. A balanced gut microbiota in domestic animals supports health, growth, and productivity. Probiotics (beneficial live microbes), prebiotics (non-digestible components that feed probiotic bacteria), and synbiotics (a combination of both) are emerging as effective dietary supplements for enhancing gut function and overall performance. Probiotics strengthen the gut barrier, suppress harmful microbes, and regulate immunity. Prebiotics selectively boost beneficial bacteria, whereas synbiotics improve the survival and activity of probiotics in the gut. Studies across species—including cows, pigs, poultry, sheep, and goats—have highlighted various benefits to using probiotics, prebiotics, and synbiotics. These additives have been shown to improve milk yield, weight gain, immune function, and meat and egg quality while reducing disease incidence and enhancing nutrient absorption. However, challenges remain in selecting effective strains, determining optimal dosages, and ensuring cost-efficiency and regulatory compliance. Despite these hurdles, probiotics, prebiotics, and synbiotics represent promising tools for improving animal welfare and farm productivity. Continued research is essential to maximize their potential and support a more sustainable and resilient livestock industry.

## Introduction

Use of the phrase “gut health” has become increasingly common in scientific literature about human and veterinary medicine during the past few decades (Colombino et al. 2021). Gut health, defined as the “condition of symbiotic balance between the intestinal tract and microbiota, where animal welfare and health are unaffected”, is regarded as one of the most significant elements affecting overall performance in domestic animals (Bindari and Gerber 2022). The gastrointestinal microbial population is composed of at least one-thousand distinct microbial species present in animal species and affects the efficiency of energy utilization by regulating energy intake, transit, conversion, and storage (Uyeno et al. 2015). Facilitating the digestion of ingested dietary substrates is the primary role of microbes residing in the gastrointestinal tract. Gut microorganisms and the host animal exhibit a symbiotic relationship centered largely on the acquisition of nutrition (Azad et al. 2018). The gut microbiota interacts extensively with the components of the host’s diet as well as the gut mucosal immune system. The gut microbiota of domestic animals and its associated metabolic products have a significantly impact on nutrient assimilation, absorption, and metabolism, which in turn affects the overall health and growth of the animal (Hussain et al. 2021).

The FAO/WHO has defined the term “probiotic” as “*live microorganisms which confer a health benefit on the host*,* when administered in adequate amounts*” (Mazziotta et al. 2023). Probiotics are primarily live, non-pathogenic microorganisms that enhance host health by outcompeting harmful microorganisms (Kulkarni et al. 2022). Probiotics prevent and control intestinal infections while also boosting animal health and productivity (Melara et al. 2022). The biological effects of probiotics are generally exerted either through modulation of the host’s innate immune system or through direct impacts upon potentially harmful microorganisms in the gut, for example by affecting toxin production by pathogens or detoxifying food components (Oelschlaeger 2010). The mode of action of probiotics involves enhancement of the epithelial barrier function, increased adhesion to the intestinal mucosa, inhibition of pathogen adhesion and exclusion of pathogenic microorganisms, the production of antimicrobial substances, and modulation of the host defense system (You et al. 2022). By generating a wide range of inhibitory substances, such as organic acids, hydrogen peroxide, and bacteriocins, probiotic organisms play a crucial role in the inhibition of potentially harmful microbes and maintenance of the balance and growth of the beneficial microbial population in the gastrointestinal tract, which is crucial for ensuring “digestive health” (Balta et al. 2021).

Gibson and Roberfroid defined the term “prebiotic” as “a non-digestible food additive that benefits the host by selectively encouraging the growth and/or activity of one or a restricted number of bacteria in the colon, hence improving host health” (Leone and Ferrante 2023). Prebiotics can undergo selective fermentation and change the makeup and activity of beneficial intestinal microflora. Prebiotics are usually carbohydrates such as fiber and oligosaccharides that are resistant to digestion in the upper gastrointestinal tract. When these prebiotics enter the colon, they act as substrates for beneficial microbes, leading to a healthier and more balanced gut microbiota (Slavin 2013). The main function of prebiotics is to alter the gut microbiota in such a way that is beneficial to the overall health and performance of the host animal, typically by enhancing the intestinal environment (You et al. 2022). Administration of prebiotics can significantly enhance feed absorption and utilization, daily body weight gain, and overall body weight gain in a variety of animals (Markowiak and Śliżewska 2018). The mode of action of prebiotics involves selective improvement of the fermentation activity of beneficial microorganisms to stimulate the release of short-chain oligosaccharides or fatty acids into the bloodstream.

Gibson and Roberfroid initially defined the term “synbiotics” as referring to “a combination of prebiotics and probiotics that improves the survival and implantation of subsistent microbial dietary supplements in the GI tract, enhances the growth and/or activates the metabolism of one or a small number of health-promoting microbes, and ultimately improves host welfare” (Gibson and Roberfroid 1995). Enhancing the survival of probiotic bacteria in the gastrointestinal tract is the primary objective of synbiotic administration. Synbiotics were developed to address potential challenges in probiotic survival in the gastrointestinal tract and exhibit both probiotic and prebiotic effects (Rioux et al. 2005). Furthermore, compared with the individual activities of probiotics or prebiotics, combining the active constituents of both in a single product could provide for more favorable results (Bengmark 2005).

This review emphasizes literature published within the past decade (2015–2025), identified mainly through PubMed and Scopus. Earlier studies were also included when they provided important historical context or foundational insights. Conference abstracts, non-peer-reviewed sources, and duplicate records were excluded, and only peer-reviewed articles published in English were considered. Several studies have examined potential effects of probiotics and prebiotics alone or in combination in domestic animals. However, the beneficial impacts on a wide range of domesticated animals remain poorly understood. Ideally, determining the effectiveness of biotic food additives requires evaluating their overall function in a single, well-designed trial across a large, defined livestock population. This review focuses primarily on current understanding regarding the combined effects of probiotics, prebiotics, and synbiotics on the growth and productivity of various domestic animals closely associated with human welfare and economic advancement.

### Current understanding of the effects of probiotic/prebiotic supplementation on performance in cattle

Compared with other growth promoters, probiotics, prebiotics, and synbiotics used in cows are relatively inexpensive, and they have been shown to increase the efficiency of nutrient utilization, which significantly lowers feeding costs (Heinrichs et al. 2003). Probiotics have several positive effects on cattle, such as increasing body weight and lowering the frequency of diarrhea. Antibiotic therapy is often implemented to reduce the frequency of scours and preserve performance in calves. However, due to safety concerns regarding antibiotic resistance and the presence of chemical residues in animal products, probiotic additives have been developed as alternatives to enhance animal health and productivity (Berge et al. 2009).

Populations of lactobacilli and bifidobacteria typically decline in cattle herds during the early stages of life (Uyeno et al. 2010). Enhancing the enteric microbiota by augmenting beneficial microorganisms is considered an effective strategy for rearing healthy calves. Early colonization of the intestinal ecosystem by LAB (lactic acid bacteria) may reduce pathogen adherence to the intestinal mucosa (Isolauri et al. 2001). A stable microbial load of *Lactobacillus* spp. has been shown to improve weight gain and immune competence in young calves (Amin and Seifert 2021). However, the efficacy of probiotic strains may differ depending on the conditions under which calves are raised. Under stressful conditions, DFMs (Direct-fed Microbials) may mitigate the risk or severity of scours resulting from disruption of the normal intestinal environment. Further research is thus needed to enhance understanding of how selected lactobacilli and bifidobacteria strains kill pathogens, antagonize their pathogenicity, and modulate the immune response to infection (Timmerman et al. 2005).

Available data have shown that probiotic supplementation is a promising alternative therapy for uterine diseases in cattle. Probiotics containing *Lactobacillus* and *Bifidobacterium* improve fertility and enhance uterine immunity. Probiotic strains of *Lactobacillus* and *Bifidobacterium* species, whether used individually or in combination, have demonstrated the potential to enhance fertility. Strategic intravaginal administration of these formulations can enhance uterine immunity, particularly during the postpartum period (Adnane et al. 2024). Additionally, probiotics alter the composition of the microbiome within the digestive system. The use of probiotics to maintain homeostasis in the rumen has the potential to enhance feed digestion, increase the production of volatile fatty acids, and optimize nitrogen flow, all of which contribute to improved milk composition and production (Nalla et al. 2022).

Prebiotics can also improve performance, feed intake, and immunological responses in cattle and aid in maintaining the gut microbial balance. Prebiotics such as oligosaccharides support the growth of lactobacilli and bifidobacteria, thereby improving the health of the host by changing the gut microbiota. However, prebiotics may not provide significant health benefits in calves that are generally healthy. Nevertheless, it is anticipated that prebiotics will be incorporated into the diets of both ruminants and non-ruminants for the foreseeable future as a means of modifying the gut microbiota and enhancing animal productivity in a sustainable fashion (Bamigbade et al. 2022). Oligosaccharides such as MOS (Mannan Oligosaccharides) and galactosyl-lactose are thought to exert specific effects in calves (Quigley et al. 2002). Galactosyl-lactose, which is generated via the enzymatic treatment of whey with beta-galactosidase, is beneficial for enhancing the growth and health of dairy calves (Quigley et al. 1997). A previous study revealed that prebiotic supplementation can potentially improve the growth of claves by modifying microbial fermentation activity. However, most prebiotics may not exert any apparent beneficial effects, and observable benefits are likely minimal when calves are generally healthy (Quezada-Mendoza et al. 2011).

The impact of cellooligosaccharide supplementation on the intestinal ecology and performance has been assessed in Holstein calves receiving either milk replacer or whole milk. Cellooligosaccharides are known to support particular microbes within the calf intestinal tract. For example, they promote the growth of butyric acid–producing bacteria and enhance the efficiency of digestion and nutrient absorption. In vivo studies have indicated that feeding cellooligosaccharides enhances daily weight gain and feed efficiency in calves during the post-weaning period. Supplementation with prebiotics can increase protein and vitamin synthesis, milk yield, and composition (El Jeni et al. 2024). In Japan, supplementing the feed of dairy cows with a potent prebiotic known as Dextrin was shown to significantly enhance milk output (Yasuda et al. 2007). Probiotics and their associated metabolites have also demonstrated efficacy in treating mastitis in dairy cattle and gastrointestinal diseases in calves (Wang et al. 2024). Prebiotics, by comparison, aid in the prevention of mastitis in cattle by increasing metabolism, stimulating the growth of beneficial gut microbes, and facilitating the removal of harmful microbes (Yu et al. 2025).

The production capacity of dairy cows can be increased by improving forage digestibility; however, this can limit energy intake. The use of probiotics that target the rumen and affect digestive processes such as cellulolysis and microbial protein synthesis has been employed to overcome this issue. The predominant type of probiotic utilized in dairy cattle is yeast (*Saccharomyces cerevisiae*). Supplementation with lactate-utilizing bacteria such as *Megasphaera elsdenii* can help mitigate acidosis in animals on high-concentrate diets by reducing ruminal lactic acid levels and stabilizing the pH (Nocek and Kautz 2006; Nocek et al. 2002). DFMs such as *Megasphaera elsdenii* and *Propionibacterium* spp. have also been administered to prevent ruminal lactate accumulation (Klieve et al. 2003). Certain strains of active dry yeast have demonstrated efficacy in elevating and stabilizing ruminal pH by promoting the growth of ciliate protozoa, which consume starch and compete with amylolytic lactate-producing bacteria. Maintaining a stable ruminal pH is beneficial in beef cattle that are fed diets high in readily fermentable materials, as such diets can alter the ruminal microbial communities and heighten the risk of ruminal acidosis (Ghorbani et al. 2002). Yeast cells supply growth factors such as organic acids, oligosaccharides, B vitamins, and amino acids, which promote microbial growth in the rumen and help stabilize ruminal pH. The positive effect of yeast supplementation on organic matter digestibility increases with the percentage of fiber in the diet, indicating that yeast supplementation enhances rumen fermentation (Chaucheyras-Durand and Durand 2010). However, at present, there is no conclusive evidence that yeast supplementation is beneficial at all times, as it varies markedly with products and may not necessarily correspond to actual dairy production (Lynch and Martin 2002).

#### Effect of probiotic/prebiotic supplementation on performance in sheep

Supplementing sheep diets with probiotics and prebiotics has been shown to enhance growth performance and feed efficiency. A study involving 40 Pelibuey × Katahdin lambs demonstrated that adding live *Saccharomyces cerevisiae* (probiotic) or MOS with β-glucan (prebiotic) to the diets improved feed efficiency by 5.6% and 6.9%, respectively, compared with the respective control group. When both supplements were combined, ADG (Average Daily Gain) increased by 10%, and feed efficiency improved by 9.5% (Estrada-Angulo et al. 2021). A study involving growth-retarded Hu lambs demonstrated that dietary probiotic supplementation increased ADG and dry matter intake. Additionally, probiotic supplementation improved antioxidant enzyme activity and increased the concentrations of growth hormone and immunoglobulin G, indicating enhanced immune function. Probiotic supplementation also favorably altered the rumen fermentation characteristics and microbial composition, contributing to better nutrient utilization (Mao et al. 2023). Another study assessed the effects of probiotics and prebiotics, both individually and combined, on lambs finished under subtropical conditions. Although individual supplementation with either probiotics or prebiotics improved feed efficiency and dietary energy utilization, combined supplementation led to a 10% increase in ADG and 9.5% improvement in gain-to-feed ratio (Estrada-Angulo et al. 2021). This suggests a synergistic effect when both supplements are used together. Furthermore, research on Barki lambs indicated that diets supplemented with either probiotics or prebiotics at 10 g/head/day enhanced growth performance, digestibility, and economic efficiency. These findings highlight the potential of such supplements to improve production outcomes in sheep.

A study examining growing lambs reported that increasing prebiotic levels up to 0.15% increased both ADG and feed efficiency. Probiotic supplementation also improved these performance metrics, though to a lesser extent than prebiotics (Shoukry et al. 2023). Overall, integrating probiotics and prebiotics into sheep diets can lead to significant improvements in growth rates, feed efficiency, and health parameters. However, the effectiveness of supplementation may vary based on factors such as the specific strains used, dosage, and environmental conditions. Therefore, supplementation strategies should be tailored to the particular needs of the flock, under the guidance of animal nutrition experts, in order to achieve optimal results.

#### Effect of probiotic/prebiotic supplementation on performance in goats

Supplementing goat diets with probiotics, prebiotics, or synbiotics has been shown to enhance growth performance, nutrient utilization, and milk production. A study involving growing goats demonstrated that adding a microbial feed supplement to a balanced diet significantly increased ADG compared with the control diet. Specifically, goats receiving the supplement gained more weight daily than those on a standard diet without supplementation. In lactating Saanen dairy goats, dietary supplementation with probiotics such as *S. cerevisiae*, *B. subtilis*, and *Enterococcus faecalis* led to increased dry matter intake and enhanced milk yield. Notably, milk fat percentage improved with mixed probiotic supplementation, whereas supplementation with individual probiotics elevated milk protein and lactose percentages (Ma et al. 2020). Research on late-lactation Murciano-Granadina goats indicated that incorporating a postbiotic yeast fermentation product into the diet improved fiber digestibility and increased ruminal propionate levels. These changes were associated with enhanced energy efficiency for milk production and a reduction in methane emissions per unit of milk produced (Fernández et al. 2023).

Feeding goats a biotic diet was shown to increase meat dressing and protein content while decreasing fat and saturated fatty acid levels and increasing the percentage of unsaturated fatty acids. Both prebiotics and probiotics can be added to goat diets to enhance meat quality and yield (Mierlita et al. 2023). Adding Jerusalem artichoke tuber extract, a powerful prebiotic, to goat diets can increase feed efficiency, enhance digestion, and lessen the incidence of diarrhea. Beneficial probiotics boost the synthesis of antimicrobial compounds in young goats and enhance epithelial adhesion and immune function (Pradhan et al. 2015). Integrating probiotics, prebiotics, or synbiotics into goat diets has an overall positive effect on growth rate, milk production, and nutrient utilization, thus contributing to improved performance and efficiency in goat production. Incorporating probiotics and prebiotics into livestock diets is a promising strategy for reducing antibiotic usage and combating antimicrobial resistance. These additives improve gut health, enhance immune function, and boost overall performance in cows, pigs, chickens, sheep, and goats. Adopting such alternatives aligns with sustainable farming practices and improves animal welfare (Odey et al. 2024).

#### Effect of probiotic/prebiotic supplementation on performance in pigs

Probiotics are generally thought to improve human health by altering the colon bacterial community, thereby preventing many illnesses. In pigs, probiotics target the colon and cecum, which harbor a diverse microbial population. Current research suggests that probiotics have a wide range of beneficial effects that have not been fully elucidated. The functional mechanisms of probiotics include modulating the gut microbiota, enhancing host immune responses and nutrient digestibility, and reducing the incidence of diarrhea, with different probiotics associated with specific modes of action (Vanbelle et al. 1990).

In pigs, antibiotics are used to promote growth and treat diseases, but due to the risks associated with antibiotic resistance, new probiotic-like nutritional additives are needed. Probiotics have been shown to enhance immune responses and improve intestinal health and nutritional efficiency. Studies have also shown that probiotics exert beneficial antimicrobial effects against pathogenic microorganisms. Probiotics improve performance during pregnancy, parturition, lactation, growth, and finishing, and their use also enhances resistance to the effects of environmental pollutants (Pereira et al. 2022). Due to their wide range of clinical benefits in pig farming, interest in nutritional strategies using probiotics is increasing. Probiotics can be administered in all stages of pig production, including in sows, neonatal piglets, early weaned piglets, and growing-finishing pigs (Yang et al. 2015a). However, standard methodologies for evaluating the benefits of probiotics in swine are lacking, as some researchers have used probiotic mixtures with unclear information regarding the exact concentrations and proportions of strains in feed rations. In swine production, by contrast, probiotics are most commonly applied during the nursery phase, especially during the period of microbiota development (Yang et al. 2015b).

Numerous studies have demonstrated the substantial impact of probiotics on immune system activation in pigs, which leads to improved health status and production metrics in fatteners and decreased diarrheal incidence and mortality rates (Samolińska et al. 2018). Administration of the prebiotic inulin prevents the growth of pathogenic and putrefactive bacteria by lowering the pH of the intestinal contents and stimulating the production of short-chain fatty acids by the beneficial gastrointestinal microbiota via enteric fermentation (Chlebicz-Wójcik and Śliżewska 2020). Weaning is a critical time during the life of pigs, as piglets experience trauma due to separation from the sow, making them more vulnerable to potentially fatal digestive disorders resulting from disruption in the balance of the gut microbiota (Nowland et al. 2021). Post-weaning stress and suboptimal growth can be alleviated by modifying the intestinal microbiota through dietary interventions. Optimizing the potentially advantageous elements of weaner diets through the addition of micro-ingredients such as probiotics, prebiotics, and synbiotics is one way to achieve the desired microbiota and enhance gut balance (Han et al. 2024) (Han et al. 2024).

Studies indicate that probiotics can boost immune function in pigs, leading to increased resistance to toxins produced by harmful bacteria; probiotics can also help stop the growth and spread of these microorganisms (Jiang et al. 2024). Oligosaccharide prebiotics are among the most important natural macromolecules for boosting immune responses and preventing infections in pigs (Gormley et al. 2024). Synbiotic-supplemented diets may boost lactate synthesis and antibody levels while inhibiting the growth of pathogenic bacteria. Pigs grow more quickly and experience less diarrhea and lower mortality risk when fed a symbiotic-containing diet (Krause et al. 2010). Pigs fed a probiotic complex containing *Bacillus* and *Saccharomyces* species showed higher body weight and daily weight gain over a 42-day period. Additionally, fecal emissions of ammonia and hydrogen sulfide were reduced. Addition of a mixed probiotic to a low-crude-protein diet was shown to improve nutrient digestibility, meat quality, and muscle area of pigs (Biswas et al. 2025). Prebiotics such as inulin and FOS (Fructo-Oligosaccharides) also enhance gut health and immune responses in pigs, contributing to better overall health and reduced antibiotic use (Liu et al. 2024).

#### Effect of probiotic/prebiotic supplementation on performance in chickens

Probiotics, prebiotics, and postbiotics are examples of “biotic” feed additives that can improve the growth and health of chickens (Saeed et al. 2023). These biotic components are nutritionally viable substitutes for growth boosters in animal feed. The use of these additives promotes the growth of broiler chickens, leading to improved meat quality (Ayalew et al. 2022). Several researchers have examined the impact of probiotic-prebiotic supplementation on broiler performance. In a study by Utami et al., 200 broiler chicks aged 35 days were divided into five groups and subjected to different treatments. Chicks that received probiotic supplementation exhibited increased body weight and decreased feed conversion, but feed consumption was not significantly affected. These results suggest that probiotic supplementation is a useful and safe way to increase meat production (Utami and Wahyono 2018).

Hens and turkeys fed a diet containing the commercial lactic acid bacteria–based probiotic FloraMax PW Boehringer Ingelheim showed greater resistance to infection with *Salmonella* sp. (Prado-Rebolledo et al. 2017). Probiotics improve performance in chickens and thus represent a potential alternative to antibiotics (Higgins et al. 2011). Moreover, probiotics decrease the risks of various infections in chickens. Murakami et al. (2024) demonstrated that administration of the probiotic *LIC37* effectively prevents *Campylobacter jejuni* infection in chicks. *LIC37* adheres well to the cecal wall and promotes the growth of beneficial microorganisms, including *Blautia* species. These findings also suggest that *LIC37* is a practical and effective probiotic agent for preventing *C. jejuni* outbreaks on poultry farms. Probiotics are a useful tool for lowering the risk of infection in chickens (Murakami et al. 2024). *LIC37* was also shown to decrease tumor necrosis factor–α and interleukin (IL)−6 levels and increase IL-10 levels, suggesting that *LIC37* exerts anti-inflammatory effects through modulation of cytokine profiles (Tsukagoshi et al. 2020). Sirisopapong et al. (2023) analyzed LAB strains from the digestive tract of chickens to evaluate their potential for use as probiotics in poultry. They isolated 2,000 colonies, from which 200 LAB strains were identified. Two probiotic strains (*Limosilactobacillus ingluviei* and *Limosilactobacillus salivarius*) were tested in vitro. Their results showed that *L. ingluviei* and *L. salivarius* increased LAB and *Bifidobacterium* populations while reducing *Enterobacteriaceae* and *Escherichia coli* populations in chicken cecal contents. These results demonstrated that probiotics exert highly beneficial effects in the digestive tract of chickens (Sirisopapong et al. 2023).

To promote farmer adoption and support broader use of commercial probiotics, formulations must be cost-effective, stable when mixed with feed (including survival of organisms during heat pelleting), shelf-stable, and compliant with regulatory standards and labeling requirements. Bacterial spore formers, which are typically of the genus *Bacillus*, are present in certain probiotic products and have been shown to prevent specific gastrointestinal issues. *Bacillus*-DFM prevents disorders of the gastrointestinal tract and provides a variety of nutritional benefits to both humans and animals (Hong et al. 2005; Sen et al. 2012; Vreeland et al. 2000). In the presence of feed, 90% of *B. subtilis* spores germinate in various segments of the gastrointestinal tract within 60 min, as demonstrated by both in vitro and in vivo investigations (Latorre et al. 2014a). The growth performance, digesta viscosity, bacterial translocation, microbiota composition, and bone mineralization of broiler chickens and turkeys fed a rye-based diet were all enhanced by the addition of *Bacillus*-based DFM (Latorre et al. 2014b). Enzyme production from the combined *Bacillus* spp. strains employed as DFM can improve intestinal integrity, boost growth performance, and enhance nutrient absorption (Latorre et al. 2015).

Prebiotics help microbes ferment nondigestible food components such as oligosaccharides to enhance gut function. Prebiotics facilitate the proliferation of bifidobacteria and lactobacilli in the intestine, thereby improving the health of the host (Hedin et al. 2007). Prebiotics have also been shown to alter the colonic microbiota and impact gut metabolism in humans. Prebiotics enhance host defense and lower pathogen-induced bird mortality (Ducatelle et al. 2015). Poultry administered prebiotics exhibit elevated populations of lactobacilli and bifidobacteria and decreased populations of clostridia and other harmful bacteria, along with improved eggshell and bone quality. *Aspergillus* meal, a commonly used prebiotic, contains beta-glucans, FOS, chitosan, and MOS, which can improve growth and immunity in chickens (Gormley et al. 2024). Beta-glucan is a potent immune booster, whereas MOS bind toxin active sites and defend the gut microbiota against invasion. FOS and chitosan are non-digestible carbohydrates readily fermented by the gut flora. Prebiotic-fed chicks have lower ileum energy and protein content, indicating greater digestion and absorption of nutrients (Jonker et al. 2010; Kim et al. 2006). Yalçın et al. (2014) reported that cell wall material derived from baker’s yeast is an effective prebiotic feed additive for broilers, enhancing growth performance and humoral immune responses and reducing abdominal obesity. Yeast cell wall material also stimulates the production of low-cholesterol eggs and enhances the humoral immunity response in laying hens. Additional research has shown that prebiotic supplementation enhances colonization of the intestinal tract by *Bacillus* spp., *Lactobacillus* sp., and *Clostridium* sp. and strengthens the epithelial barrier function. Additionally, colonization by pathogenic bacteria (*E. coli* and *Salmonella* spp.) is reduced. Prebiotics provide energy, metabolic substrates, and essential micronutrients to broiler chickens, thereby improving growth and increasing the feed conversion ratio. Prebiotic supplementation reduces the incidence of various diseases by inhibiting colonization of the gut lining by pathogenic bacteria and reducing the intestinal pathogen count, enhancing immune function, and improving gut morpho-functional characteristics, thus enhancing overall broiler health (Rosen 2007). Consequently, these feed additives enhance growth performance by promoting the recovery of gut health and digestive functions that have been compromised by stress (Kridtayopas et al. 2019).

In laying hens, prebiotic supplementation of feed improves productivity and increases eggshell thickness and calcium retention. The potential of prebiotics to lower the cholesterol level in egg yolk has attracted considerable attention (Shehata et al. 2022). Prebiotics such as fermentable sugars and exogenous enzymes are utilized as feed additives to enhance host health and shield chickens against infectious bacteria (Hashem et al. 2022). Prebiotic administration enhances the gut microenvironment by increasing the number of good bacteria, thereby inhibiting intestinal colonization by pathogens (Ji et al. 2023). By supporting a balanced and healthy gut microbiota, both probiotics and prebiotics can improve the overall resilience of the intestinal ecology and reduce the vulnerability to colonization by pathogens (Han et al. 2017). Moreover, probiotics are effective for modulating the gut microbiota, enhancing immune function, and improving growth performance in poultry (Markowiak and Śliżewska 2018). Probiotics aid in suppressing colonization by pathogens such as *Salmonella* spp. and *Clostridium perfringens*, leading to decreased disease incidence and antibiotic usage. Prebiotics, including MOS and FOS, support the growth of beneficial bacteria and improve gut health in poultry production, further reducing the need for antibiotics (Bilal et al. 2023).

#### Effect of probiotic/prebiotic supplementation on performance in rabbits

The use of probiotics in rabbits has been shown to improve growth performance and feed efficiency, particularly under conditions of heat stress. Some studies suggest that adding strains such as *Bifidobacterium bifidum*, *Lactobacillus acidophilus*, *Clostridium butyricum*, or *Saccharomyces cerevisiae* can improve final body weight, ADG, and FCR (Feed Conversion Ratio) in rabbits. Probiotics can also increase villus height and decrease crypt depth, thereby enhancing the villus-to-crypt ratio in the small intestine, which supports improved nutrient absorption (Ashour et al. 2024). *Clostridium butyricum* has additionally been associated with improved gut health, antioxidative properties, immune responsiveness, and overall growth performance (Liu et al. 2019). Other strains, including *Lactobacillus acidophilus*,* L. casei*,* L. plantarum*,* L. rhamnosus*,* Enterococcus faecium*, and *B. bifidum*, have demonstrated beneficial effects on growth, feed efficiency, gut microbiota balance, and health; however, the magnitude of these effects varies depending on strain, dosage, and management conditions (Mancini and Paci 2021). Supplementation of breastfeeding does and their kits with probiotics has been shown to significantly enhance immune and antioxidant responses, modulate gut microbiota composition, and improve production performance (Zhao et al. 2025). However, not all trials have found benefits, as some strain combinations show no effect or even antagonism when combined, suggesting that impacts depend strongly on the probiotic type, dose, and life stage of the target animal (Fathi et al. 2017). Prebiotics alone can improve growth and carcass weight, as FOS at levels such as 0.5–1% in drinking water were shown to significantly improve body weight gain and lower FCR in New Zealand White and APRI rabbits compared with controls.

Additionally, carcass yield and dressing percentage increased, whereas pathogen counts dropped, and beneficial bacteria, such as *Lactobacillus*, increased (Abd El-Aziz et al. 2022). FOS also boosted antioxidant markers and improved serum protein levels without harmful effects on liver or kidney function. In a 12-week trial comparing Biotronic prebiotic vs. Biovet^®^ probiotic vs. symbiotic, the prebiotic alone led to significantly higher final body weight and ADG, with improved FCR (Nwachukwu et al. 2021). Symbiotics (a combination of probiotics and prebiotics) can also enhance growth performance, lipid profiles, and gut histology in hot climates. However, concerns exist regarding the unnecessary use of probiotics, which can disrupt the native gut flora and potentially lead to excessive gas, gastrointestinal stasis, or diarrhea. High-quality hay and diet are often more critical than supplements for healthy rabbits (Nwachukwu et al. 2021).

In conclusion, supplementation with prebiotics reliably enhances growth performance and feed efficiency in rabbits, whereas probiotics alone yield more variable results, depending on the strain and dosage. Combining the two into synbiotics often produces the most consistent gains, particularly under conditions of environmental stress. Other effects of probiotic/prebiotic supplementation are summarized in Table 1, which outlines the numerous beneficial effects of probiotics in animal health.

**Table 1:** Recent probiotic/prebiotic trials in various livestock animals

Animal	Probiotic/Prebiotic/Synbiotic	Positive Effects	Refs.
Cows	*Lacticaseibacillus casei* ssp. *casei* JCM1134	Improves growth rate and general health parameters	(Hasunuma et al. 2011)
	*Faecalibacterium prausnitzii*	Decreases diarrhea incidence and supports intestinal equilibrium	(Foditsch et al. 2015)
	*Saccharomyces cerevisiae boulardii* CNCM I-1079	Stimulates immune function in the gastrointestinal tract	(Villot et al. 2020)
	*Lactiplantibacillus plantarum* ATCC11095 *Enterococcus faecalis* *Saccharomyces cerevisiae* *Aspergillus niger* CICC2377	Improves rumen enzyme activities and up-regulates the levels of glycerophospholipids in the rumen	(Zhang et al. 2025)
	*Limosilactobacillus reuteri* DSM17938	Promotes in situ vitamin B synthesis within the gastrointestinal tract	(Luo et al. 2022)
	*Enterococcus mundtii* H81	Increases resistance to mastitis	(Qiu et al. 2022)
	*Lactiplantibacillus plantarum* DSM26912	Exhibits antimicrobial activity against *Staphylococcus aureus*	(Titze and Krömker 2020)
	*Lacticaseibacillus mucosae* CRL2069	Alleviates intestinal inflammation via TLR pathway modulation	(Mansilla et al. 2020)
Pigs	*Lactiplantibacillus plantarum* PFM 105	Improves intestinal morphology and microbial balance in weaned piglets	(Wang et al. 2019)
	*Pediococcus acidilactici* GB-U15 *+* lactulose	Modulates gut microbiota, strengthens intestinal immunity	(Guevarra et al. 2022)
	*Rhodopseudomonas sphaeroides* *Saccharomyces cerevisiae* *Bifidobacterium bifidum*	Promote the growth performance of piglets by activating the JAK2/STAT5 signaling pathway	(Li et al. 2025)
	*Lacticaseibacillus paracasei* ŁOCK 1091 *Lactiplantibacillus pentosus* ŁOCK 1094 *Lactiplantibacillus plantarum* ŁOCK 0860 *Limosilactobacillus reuteri* ŁOCK 1092 *Lacticaseibacillus rhamnosus* ŁOCK 1087 *Saccaromyces cerevisiae* ŁOCK 0119 *+* Inulin	Reduce the occurrence of diarrhea	(Chlebicz-Wójcik and Śliżewska 2020)
	*Enterococcus faecium* *Bacillus subtilis* *Saccharomyces cerevisiae*	Promotes weight gain and improves microbial diversity in the gut	(Park et al. 2024)
	*Limosilactobacillus reuteri*	Reduce colitis	(Xu et al. 2025)
	*Lactiplantibacillus plantarum* *Bacillus subtilis* *Saccharomyces cerevisiae* + yeast cell wall +β-glucans +glyconutrients	Enhances growth, improves fatty acid profile, and provides for superior meat quality	(Sampath et al. 2023)
Poultry	*Pediococcus acidilactici*	Increases egg production and improves feed conversion ratio	(Mikulski et al. 2020)
	*Bacillus subtilis*, *Bacillus licheniformis* *Clostridium butyricum*	Enhances egg production and lowers cholesterol content in egg yolks	(Saleh et al. 2024)
	*Limosilactobacillus ingluviei* C37	Inhibits colonization by *Campylobacter jejuni*	(Murakami et al. 2024)
	Galactooligosaccharides	Suppresses *Salmonella* spp. colonization	(Pourabedin and Zhao 2015)
	*Lactobacillus acidophilus* + Mannan oligosaccharides	Improves growth performance, enhances meat quality	(Dev et al. 2020)
	*Enterococcus faecium* *Bacillus subtilis* *Saccharomyces cerevisiae* *Lactiplantibacillus plantarum* + ginseng polysaccharides	Improving intestinal morphology and microbial composition	(Xie et al. 2020)
Sheep	*Lactobacillus acidophilus*	Stimulated feed intake and daily weight gain	(Mao et al. 2023)
	*Lactobacillus johnsonii* M5	Enhances immunity and antioxidant capacity, improving intestinal health	(Wang et al. 2025)
	*Lactobacillus acidophilus* INMIA 9602	Reduce *Salmonella* infection	(Pepoyan et al. 2020)
	*Bacillus amyloliquefaciens* +Mannan-oligosaccharides + β-glucans *+* oligomeric isomaltose	Enhances average daily gain and feed efficiency	(Shoukry et al. 2023)
Goats	*Enterococcus faecium* *Bacteroides fragilis*	Reduce diarrheal	(Essa et al. 2025)
	*Debaryomyces hansenii* CBS 8339	Induce innage immune memory	(Angulo and Angulo 2025)
	*Saccharomyces cerevisiae* *Bacillus subtilis* *Enterococcus faecalis*	Increases dry matter intake and milk yield Improves milk composition	(Ma et al. 2020)
	*Bacillus amyloliquefaciens* fsznc-06 *Bacillus pumilus* fsznc-09	Enhances the abundance of beneficial gut microbes and intestinal structural development	(Zhang et al. 2020)
Rabbits	*Bifidobacterium bifidum* *Lactobacillus acidophilus*	Increases feed conversion ratio and growth performance	(Ashour et al. 2024)
	*Clostridium butyricum*	Increases growth performance Enhances intestinal immune response	(Liu et al. 2019)
	*Bacillus subtilis* *Bacillus licheniformis* *Saccharomyces cerevisiae*	Improves the immune system, antioxidant capacity, and lactation performance	(Zhao et al. 2025)
	*Bacillus subtilis*	Increases hemoglobin, red blood cells, and platelets	(Fathi et al. 2017)
	Fructooligosaccharide	Improves growth performance, carcass features, hematological parameters, antioxidant status, and cecal microbiota in	(Abd El-Aziz et al. 2022)
	*Saccharomyces boulardii* *Lactobacillus acidophilus* *Saccharomyces cerevisiae* +Fructooligosaccharide	Improves intestinal development, blood profiles, and aid in feed digestion, nutrient absorption	(Nwachukwu et al. 2021)

### Common mechanisms and benefits across species

Probiotic microorganisms such as *Lactobacillus* spp., *Bifidobacterium* spp., *Enterococcus* spp., *Bacillus subtilis* spp., and *Saccharomyces cerevisiae* produce bacteriocins, organic acids, hydrogen peroxide, and quorum-sensing inhibitors that reduce pathogens such as *Salmonella*, *E. coli*, *Clostridium*, and enterotoxigenic *E. coli*. Probiotics compete for adhesion sites and nutrients, thus establishing colonization resistance and lowering pathogen load comparably to AGPs (Antibiotic Growth Promoters) in poultry and swine (Zhang et al. 2023). Prebiotics such as MOS, FOS, and β-glucans serve as bacterial decoys that support beneficial microbial colonization. These biotics are integrated into national and global AGP bans and now serve as mainstream non-antibiotic growth promoters without contributing to antimicrobial resistance (Ji et al. 2023).

Probiotics enhance immune modulation and barrier reinforcement, boosting phagocytic activity, oxidative burst activity, adaptive responses, gut-associated lymphoid tissue development, and barrier function. Synbiotic synergy, which combines probiotics with matching prebiotics, consistently outperforms individual components across species, improving probiotic viability/colonization and targeted substrate utilization by the commensal microbiota (Hardy et al. 2013).

### Challenges and limitations

Probiotics and prebiotics have several disadvantages with regard to effectiveness, including strain-specific responses, stability and viability issues, regulatory and safety considerations, economic and practical constraints, limited understanding of mechanisms, and potential adverse effects. The efficacy of a probiotic depends on the specific microbial strain used, host factors, and the species, age, health status, and gut microbiota composition of the animal species. Stability and viability issues include processing sensitivity, shelf-life limitations, regulatory variability, and economic constraints. The use of probiotics in animal agriculture faces economic barriers due to the relatively higher cost compared with antibiotics, in addition to issues related to regulation and production overhead costs. Animal trials showed that probiotic-containing feeds achieve a higher benefit-to-cost ratio, but only modest margin gains. Smaller farms face higher per-unit costs and variable efficacy, which discourages consistent use among farmers.

**Fig. 1:**
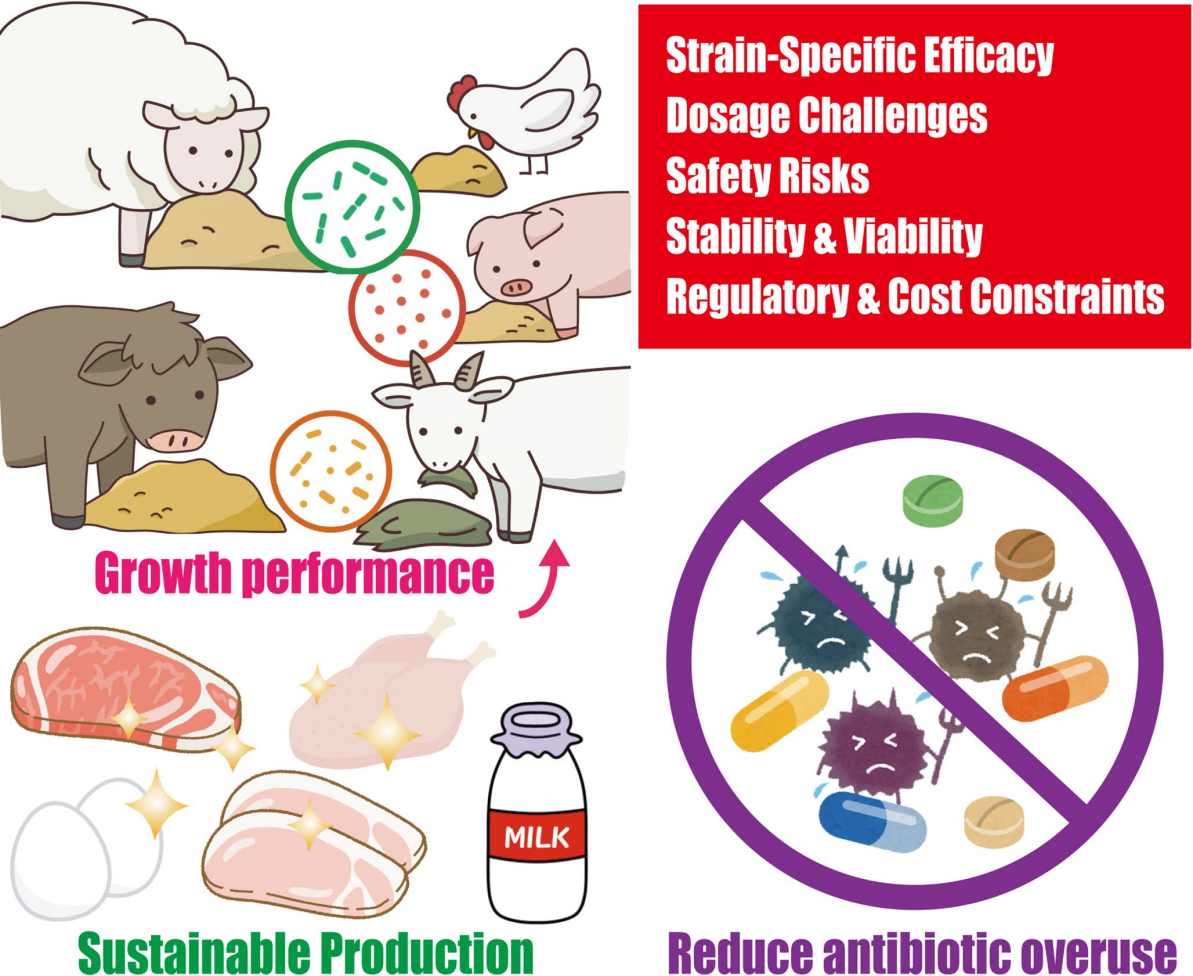
Key challenges in the application of probiotics and prebiotics in livestock production. Limitations include strain-specific efficacy, as outcomes vary with microbial strain, and dosage optimization due to the lack of standardized protocols. Safety concerns—such as virulence factors and antibiotic resistance genes—require rigorous evaluation. Stability and viability issues during processing and storage further complicate use. Regulatory complexity and economic barriers also hinder widespread adoption. Addressing these challenges is essential to achieve full realization of the potential of probiotics and prebiotics for improving animal health, enhancing growth performance, and reducing antibiotic dependence, ultimately contributing to more sustainable and efficient livestock production systems

Safety assessments are essential for preventing the spread of antibiotic resistance genes and opportunistic infections. Economic and practical constraints include low cost-to-benefit ratio, implementation challenges, and limited understanding of the mechanisms by which probiotics affect animal growth and health. Health risks, such as infections or metabolic disturbances, are also a concern (Fig. 1). Future research should focus on strain selection, understanding host-microbiome interactions, product stability, and safety assessments to optimize the benefits of probiotics in animal agriculture. Probiotics in which organisms harbor antibiotic resistance genes could potentially transfer these genes to pathogenic bacteria in the animal gut or in the environment, thereby contributing to the spread of antibiotic resistance. Long-term studies in both animals and humans are needed to assess the effectiveness and safety of probiotics, particularly in vulnerable populations.

Probiotic strains may exhibit unwanted traits, such as the expression of virulence factors, transferable antibiotic resistance, hemolytic potential, and the generation of harmful biochemicals, similar to other organisms. The effects of probiotics vary according to strain (FAO/WHO 2001). Therefore, it is crucial to carefully choose strains for specific animal applications. Determining the optimal dosage of a probiotic or prebiotic is also challenging. Dosage can vary based on the desired positive effect; therefore, different benefits may require different levels of probiotics/prebiotics. The expense of probiotics or prebiotics is becoming problematic because profit margins are getting smaller in animal husbandry. The regulatory framework for biotic food additives is also intricate. Different jurisdictions allow differing degrees of health claims in human applications. Probiotic and prebiotic manufacturers must submit experimental proof of a product’s identification, safety, and effectiveness, which are evaluated by an expert committee.

## Conclusion and future directions

In conclusion, the integration of probiotics and prebiotics into the diets of domestic animals has demonstrated potential benefits in terms of enhancing growth performance, feed efficiency, and overall health. Numerous studies have demonstrated improvements across a variety of species, including increased weight gain in pigs and broiler chicks, as well as enhanced milk yield and quality in dairy cows and sows. Prebiotics can be used in place of probiotics or to enhance their effectiveness. Interestingly, the use of components exhibiting synergistic effects may be even more efficient for stimulating the gut microbiota and protecting animal health. Supplementation with probiotics and prebiotics in rabbits has been shown to enhance growth performance, feed efficiency, and gut health by improving nutrient absorption and modulating the intestinal microbiota. These effects collectively contribute to better productivity and reduced reliance on antibiotics in rabbit production systems.

This review highlighted the significant potential of probiotics and prebiotics for enhancing the growth, productivity, and overall health of various domestic animals, including cows, pigs, chickens, sheep, goats and rabbits. Table 1 summarizes the findings of published research. These probiotics, prebiotics and synbiotics have been shown to improve nutrient utilization, immune function, gut microbiota balance, and disease resistance, ultimately leading to better animal performance and economic benefits for livestock producers. Probiotics and prebiotics work synergistically to promote a healthy gut environment, which is crucial for optimal digestion, nutrient absorption, and overall animal welfare. However, the magnitude and consistency of these effects remain variable and are strongly influenced by factors such as microbial strain specificity, animal species, age, diet composition, environmental stressors, and management practices. The current literature is also limited by a lack of long-term, large-scale, and species-specific trials, making it difficult to establish standardized recommendations across production systems. Additionally, challenges related to product stability, regulatory frameworks, and economic feasibility pose further limitations to their widespread adoption.

Synbiotics have been found to outperform individual prebiotic or probiotic treatments in various livestock species, enhancing growth, nutrient digestibility, feed efficiency, antioxidant status, and immune markers. This is evident in broilers, in which probiotic-supplemented regimens were shown to improve rumen microbial diversity, humoral immunity, and growth performance under barn feeding regimes. However, data regarding synbiotics in goats remain sparse, with most studies focusing on probiotics alone rather than fully formulated synbiotic blends. Additionally, mechanistic studies linking synbiotics to host transcriptomics, metabolomics, and microbiome ecology in goats are limited. The optimal pairing of probiotic strains with specific goat feeds and corresponding prebiotics is not yet established.

The selection of appropriate strains, the determination of optimal dosages, and ensuring safety and efficacy remain critical considerations. Additionally, the cost of these additives and the regulatory complexities surrounding their use pose further obstacles to their widespread adoption in animal husbandry. Future research should prioritize well-designed studies that address these limitations, with particular emphasis on clarifying optimal strains, dosages, and combinations of probiotics, prebiotics, and synbiotics. Emerging frontiers, such as the application of omics technologies, hold great promise for elucidating host–microbiota interactions and identifying novel functional strains tailored to specific livestock needs. The development of genetically engineered probiotics and the concept of personalized probiotic/prebiotic formulations based on an animal’s baseline microbiota represent additional exciting directions. From an industry perspective, adoption will require not only scientific validation but also greater efforts to standardize product quality, ensure safety, and harmonize regulatory frameworks. When integrated into sustainable feeding strategies, these biotic additives could contribute to reducing antibiotic dependence, improving animal welfare, and enhancing productivity in a cost-effective and environmentally responsible manner.

## Data Availability

The datasets generated during the current study are available from the corresponding author upon reasonable request.
